# Genotypic variation in Na, K and their ratio in 45 commercial cultivars of Indian tropical onion: A pressing need to reduce hypertension among the population

**DOI:** 10.3389/fnut.2023.1098320

**Published:** 2023-02-21

**Authors:** Hira Singh, Mauro Lombardo, Abhishek Goyal, Amrender Kumar, Anil Khar

**Affiliations:** ^1^Department of Vegetable Science, Punjab Agricultural University, Ludhiana, India; ^2^Division of Vegetable Science, ICAR-Indian Agricultural Research Institute, New Delhi, India; ^3^Department of Human Sciences and Promotion of the Quality of Life, San Raffaele Open University, Rome, Italy; ^4^Department of Cardiology, Dayanand Medical College and Hospital, Ludhiana, India; ^5^Agricultural Knowledge Management Unit, ICAR-Indian Agricultural Research Institute, New Delhi, India

**Keywords:** *Allium cepa* L., cardiovascular diseases, hypertension, onion bulbs, potassium, potassium/sodium ratio, sodium

## Abstract

The intake of diets with higher sodium (Na) and lower potassium (K) has been considered a leading factor for the development of hypertension (HTN). Majority of junk, processed and packaged food have higher Na contents. To counter the effects of diet on HTN, the identification of high K/Na ratio plant-based food is needed. Among fruits and vegetables, onion could be the ideal option since it contains high K content. Keeping this in mind, 45 commercially well adapted short day Indian onion cultivars were evaluated for K and Na content and their ratio to isolate suitable cultivars to prevent HTN in the Indian population. The data suggested wide variation among the genotypes for K, Na, and K/Na ratio ranging from 490.2 ± 17.0 to 9160.0 ± 96.7 mg/kg on dry matter basis, 52.7 ± 3.0 to 458.2 ± 61.7 mg/kg on dry matter basis and 3.1 ± 0.7 to 109.5 ± 17.3, respectively. The K content was recorded as significantly highest in the yellow-coloured bulb variety “Arka Pitamber” (9160.1 ± 96.7) followed by Pusa Sona (7933.2 ± 292.8). On the other hand, minimal K was assessed in the white-coloured bulb variety “Agrifound White” (490.3 ± 17.0) followed by Udaipur Local (732.9 ± 93.4). Twelve cultivars exhibited > 7000 mg K content, while nine cultivars recorded < 1500 mg. On the contrary, Na was recorded as significantly highest in the dark-red-coloured bulbs and the lowest in white bulbs. Furthermore, it was determined that there was a more than 35-fold difference observed between the highest (109.5) and lowest (3.1) K/Na ratio in the bulbs of tested cultivars. Cluster analysis revealed three major groups comprising of 23, 13 and 9 genotypes. This information could form the base for public health, food and onion researchers to design suitable cultivars to prevent HTN as a population-wide approach. The next century is going to be food-based for the amelioration of human diseases in a sustainable way without any after-effects on the human body.

## 1. Introduction

Globally, non-communicable diseases (NCDs) are becoming the foremost cause of death. Approximately 80% of deaths due to NCDs occur in countries with low to middle incomes (1). Principally, NCDs consisting of cardiovascular diseases (CVDs), diabetes, various types of cancer and chronic lung dysfunction are responsible for the majority of deaths. According to one of the estimates in 2010, about 1.39 billion individuals (31.1%) in the adult population were suffering from hypertension (HTN); this number has been constantly increasing since then. Therefore, HTN has now become a major health issue worldwide (2). Among the various factors causing NCDs and metabolic disorders, unhealthy diets are a prominent cause of HTN. According to WHO observations, people from low to middle income countries consume table salt much more than is recommended (1). According to a previous study, global mean sodium intake was quite high (3.95 gm per day) in 2010 compared to the recommended intake (< 2.3 gm per day) in major published guidelines.

Onion (*Allium cepa* L., 2*n* = 2× = 16), a bulbous vegetable and condiment crop, belonging to the *Amaryllidaceae* family, is one of the most important crop which has been domesticated and cultivated worldwide for more than 5000 years due to their peculiar properties as food, their therapeutic value and ethnopharmacological properties. This crop is grown in all climates worldwide (3–6) and is the third most important horticultural crop after potato and tomato (7). Its bulbs are an enriched source of various health promoting phytochemicals and nutrients and this crop has an utmost valorisation globally due to its multifarious uses in every community and society across the globe. The Queen of French cuisine, Julia Child, stated: “It is hard to imagine a civilisation without onions.” Every community, region and country have various traditional and folk remedies but there is a great need to document them in a systematic way to form the foundation of more scientific and modern research on that particular aspect.

The World Health Organization (WHO) also endorses the use of fresh onion extracts for treating colds, coughs, bronchitis, asthma, and appetite loss, as well as relieving hoarseness and preventing atherosclerosis (8). Because of its naturally possession of higher amounts of flavonoids and widely popular across the world, the onion crop became an interesting and fascinating vegetable (9, 10). Onions are recommended to lighten blood and lymph stagnation and to improve sexual debility or weakness. Regularly taken on an empty stomach, a mixture of white onion and honey was considered as an exceptional aphrodisiac tonic (11). Being the leading country in onion production, Indian farmers harvested 26.7 million tons from 1.4-million-hectares (12).

The 21st century is going to work on the principle of “Food as Medicine” and onion will surely play a large role in this. Since antiquity, the bulbous onion has played an important role in human health as it is being used in every kitchen in India. Most of the breeding experiments focused only on enhancing yield and yield-attributing components. However, little focus has been given to improving various quality characteristics. In Indian onions, not much scientific data on nutritional properties are available (13). Onion bulbs are enriched with potassium, vitamin C, folic acid and dietary fibre, also possessing good amounts of iron and calcium; however, they are lower in sodium and fat (8, 14–16). In USA onion cultivars, Metrani et al. (17) quantified 13,550.1 mg/kg potassium in red onion bulbs.

The comprehensive information of the genotypic difference in the potassium and sodium concentration in onion bulbs could be an epitomized contribution for people who are suffering from HTN and prone to CVD. The ratio of potassium and sodium across the genotypes varies with bulb colour and geographical location, which may support the development of future cultivars which are nutrition- and disease-specific.

HTN and CVD may be the result of metabolic syndrome; this has received the global attention of nutrition and health researchers (18). Potassium and sodium are the most important elements, being essential for normal and proper cellular functioning in the body. As it enhances the risks of high blood pressure, HTN (19, 20), CVD (21, 22), and obesity (23, 24) higher sodium and lower potassium dietary intake has become a serious global health challenge. Global research reports revealed that adverse ratio of both electrolytes is strongly linked to blood pressure (20, 21, 25). It is well documented that the dietary Na:K ratio is an independent risk factor for metabolic syndrome. Furthermore, it was suggested to modify ratios, including lower Na intakes and higher K intakes, to prevent metabolic disorders (18).

Keeping this in mind, the present study was conducted with the aim (a) to evaluate the potential cultivars representing diverse bulb colours and geographical locations with higher available potassium levels for utilisation in future onion breeding programs, (b) to identify the genotypes of Indian onions possessing the lowest sodium content in their bulbs, and (c) to select cultivars exhibiting higher potassium and sodium ratios for the regulation of blood pressure in hypertensive people.

## 2. Materials and methods

### 2.1. Location and climate

This experiment was carried out in the Division of Vegetable Science, ICAR-Indian Agricultural Research Institute, New Delhi, which is situated at 28.63*^o^*N latitude and 77.15*^o^*E longitudes and a mean height of 228 m above mean sea level. This geographical location falls in the *Trans*-Gangetic agro-climatic zone of India.

### 2.2. Plant material

A total of 45 different commercially grown varieties (Table 1) comprising different bulb colours from white to dark red were collected from different states of the country (representing more than 10 onion-producing states) and evaluated. Seeds of all varieties were maintained and produced during 2019–2020 at the Vegetable Research Farm, Division of Vegetable Science, IARI, New Delhi. After proper cleaning, harvested seeds were stored under ambient conditions. In October 2020, fungicide-treated seeds were sown for nursery production. After 6–7 weeks, the seedlings of all genotypes were transplanted in January 2021. All of the agronomical packages and practices recommended by the IARI for raising successful bulb crops were followed. This experiment was laid out in a Randomised Block Design, with three replications. Each replication included about 200 plants per plot of each variety.

**TABLE 1:** List of open pollinated short day commercial Indian onion varieties used for assessment of bioactive compounds.

S. No	Variety	Code	Bulb colour	Institute/ University	Releasing state	Country region
1	Akola Safed	AKLS	White	IARI	Maharashtra	W
2	Early Grano	EG	Yellow	IARI	New Delhi	N
3	Bhima Shubra	BSBR	White	DOGR	Maharashtra	W
4	JWO-1	JWO1	White	JAU	Gujarat	W
5	Bhima Shweta	BSWT	White	DOGR	Maharashtra	W
6	Pusa Riddhi	PRDI	Red	IARI	New Delhi	N
7	Pusa White Round	PWR	White	IARI	New Delhi	N
8	NHRDF Fursungi	NFRS	Red	NHRDF	Maharashtra	W
9	PKV White	PKVW	White	NHRDF	Maharashtra	W
10	Bhima Shakti	BSKT	Red	DOGR	Maharashtra	W
11	Pusa White Flat	PWF	White	IARI	New Delhi	N
12	VL Pyaz	VLPZ	Red	VPKAS	Uttarakhand	N
13	RO-252	R252	Red	RAU	Rajasthan	N
14	Udaipur Local	ULCL	Red	RAU	Rajasthan	N
15	GJWO-3	GJW3	White	JAU	Gujarat	W
16	GJWO-11	GJ11	White	JAU	Gujarat	W
17	JNDWO-085	JNW8	White	JAU	Gujarat	W
18	Arka Pitamber	APTB	Yellow	IIHR	Karnataka	S
19	Bhima Kiran	BKRN	Red	DOGR	Maharashtra	W
20	Phursungi Local	PHLC	Pink	NHRDF	Maharashtra	W
21	Pusa Shobha	PSOB	Brown	IARI	New Delhi	N
22	Agrifound White	AFW	White	NHRDF	Maharashtra	W
23	Pusa Sona	PSON	Yellow	IARI	New Delhi	N
24	Talaja Red	TZRD	Red	JAU	Gujarat	W
25	JRO-11	JR11	Red	JAU	Gujarat	W
26	Bhima Raj	BRAJ	Red	DOGR	Maharashtra	W
27	HOS-4	HOS4	Red	CCSHAU	Haryana	N
28	Bhima Light Red	BLRD	Red	DOGR	Maharashtra	W
29	Pusa Madhavi	PMDV	Red	IARI	New Delhi	N
30	Arka Bheem	ARBM	Red	IIHR	Karnataka	S
31	NHRDF Red-4	NRD4	Red	NHRDF	Maharashtra	W
32	L-819	L819	Red	NHRDF	Haryana	N
33	Punjab Naroya	PBNR	Red	PAU	Punjab	N
34	Bhima Super	BSPR	Red	DOGR	Maharashtra	W
35	Hisar-2	HSR2	Red	CCSHAU	Haryana	N
36	B-780	B780	Red	MPKV	Maharashtra	W
37	Bhima Safed	BMSF	White	DOGR	Maharashtra	W
38	Pusa Red	PRED	Red	IARI	New Delhi	N
39	PRO-6	PRO6	Red	PAU	Punjab	N
40	Kalyanpur Round Red	KRR	Red	CSAUAT	Uttar Pradesh	N
41	Sukhsagar	SSR	Red	LOCAL	West Bengal	W
42	Bhima Dark Red	BDR	Red	DOGR	Maharashtra	W
43	RO-59	RO59	Red	RAU	Rajasthan	N
44	NHRDF-Red L-28	NL28	Red	NHRDF	Haryana	N
45	XP Red	XPR	Red	Local	New Delhi	N

### 2.3. Estimation of potassium and sodium content

Replication-wise, fully dried samples of the edible portion of the bulb were homogenised using a pestle and mortar. Half a gram of powdered sample (three replications) was taken for digestion in 20 ml of an acid solution of nitric acid (HNO_3_) and 4-perchloric acid in the ratio of 9:4 and placed in a 500 ml conical flask. The corresponding mixture was kept overnight and was placed on a hot plate the next morning for digestion until white fumes had appeared for about 2 h. After digestion, the clear solution was diluted with double-distilled autoclaved water up to 100 ml. After dilution, the mixture was filtered with Whatman Filter Paper Number-1. An Atomic Absorption Spectrophotometer (Model AA-6880, Shimadzu, Japan) was used to measure absorbance and calculate sodium and potassium contents (Table 2). Air acetylene gas was used for this study. Each sample was measured twice (*n* = 6 for each variety, 3 replications and two replicates) to avoid any handling mistakes.

**TABLE 2:** Details of autosampler atomic absorption spectrophotometer parameters.

Element	Symbol	Burner height (mm)	Wavelength for OD value (nm)	*R*-value
Potassium	K	7	766.4	0.95
Sodium	Na	7	588.0	0.94

### 2.4. Statistics

Analysis of variance (ANOVA), box plot analysis, DMRT and cluster analysis was calculated by the use of SAS software version 9.3 (SAS Institute, Cary, NC, USA). For cluster analysis, hierarchical clustering technique was used and calculating the distance between the two clusters, a complete linkage algorithm was used which works on the principle of distant neighbours or dissimilarities.

## 3. Results

### 3.1. Potassium (K) content (mg/kg of DWB)

A wide genotypic variation in K concentrations was recorded in the onion bulbs (Table 3). The average potassium content in onion bulbs was recorded to be 4679.3 mg/kg on DWB (dry weight basis), whereas it ranged from 490.3 to 9160.1. It was determined that there was a more than 18-fold difference between the highest and lowest potassium contents in the bulbs of tested onion cultivars. It was also observed that bulb colour impacted K concentration.

**TABLE 3:** Estimation of potassium (K) and sodium (Na) of fresh bulbs on dry weight basis in Indian onion varieties.

S. No	Variety name	Potassium (mg/kg)	Sodium (mg/kg)
1	Arka Pitamber	9160.1 ± 96.7^a^	120.9 ± 13.8^mnopq^
2	Pusa Sona	7933.2 ± 292.8^b^	73.8 ± 13.9^opq^
3	GJWO-11	7875.9 ± 572.8^bc^	341.0 ± 31.6^de^
4	PRO-6	7690.0 ± 188.2^bcd^	244.2 ± 13.9^fghi^
5	VL Pyaz	7660.5 ± 68.7^bcd^	347.8 ± 39.8^de^
6	Bhima Safed	7514.5 ± 181.8^bcde^	292.8 ± 28.8^ef^
7	Pusa Red	7467.1 ± 252.7^bcde^	379.7 ± 11.9^bcd^
8	Punjab Naroya	7428.6 ± 332.9^bcde^	205.6 ± 38.3^hijk^
9	Bhima Dark Red	7258.5 ± 155.2^bcde^	181.9 ± 17.9^ijklm^
10	Pusa Riddhi	7245.7 ± 236.5^bcde^	103.4 ± 11.9^nopq^
11	Kalyanpur Round Red	7214.3 ± 130.2^bcde^	279.0 ± 46.7^efg^
12	Bhima Super	7186.6 ± 106.4^bcde^	303.8 ± 39.5^ef^
13	Pusa White Round	6942.4 ± 220.8^bcde^	202.9 ± 29.4^hijkl^
14	Pusa Madhavi	6796.3 ± 433.5^bcde^	298.0 ± 39.3^ef^
15	Sukhsagar	6621.9 ± 71.3^bcde^	280.6 ± 41.3^efg^
16	XP Red	6539.3 ± 183.4^bcde^	446.8 ± 17.6^ab^
17	Bhima Kiran	6485.3 ± 145.3^cde^	147.0 ± 12.9^jklmno^
18	Akola Safed	6340.8 ± 135.1^de^	68.8 ± 4.2^pq^
19	NHRDF-Red L-28	6333.3 ± 411.3^de^	458.2 ± 61.7^a^
20	HOS-4	6322.5 ± 284.8^de^	202.2 ± 12.4^hijkl^
21	RO-252	6321.8 ± 125.6^de^	421.9 ± 16.9^abc^
22	Pusa White Flat	6142.1 ± 168.3^e^	395.2 ± 43.2^abcd^
23	Bhima Light Red	4831.7 ± 69.7^f^	129.2 ± 15.0^lmnop^
24	Pusa Shobha	4681.3 ± 109.9^fg^	236.5 ± 21.5^fghi^
25	Arka Bheem	4017.2 ± 148.6^fgh^	291.6 ± 21.3^ef^
26	B-780	3647.7 ± 88.0^fghi^	352.6 ± 28.9^cde^
27	JNDWO-085	3429.2 ± 251.3^ghi^	382.4 ± 11.2^bcd^
28	NHRDF Fursungi	3368.3 ± 159.9^hi^	183.3 ± 11.2^ijklm^
29	JRO-11	3279.0 ± 10.3^hi^	70.2 ± 8.7^pq^
30	Hisar-2	3207.1 ± 119.9^hi^	92.7 ± 11.9^nopq^
31	L-819	3035.5 ± 161.3^hi^	119.3 ± 12.7^mnopq^
32	Bhima Shakti	2529.2 ± 142.8^ij^	306.3 ± 32.9^ef^
33	Phursungi Local	1740.7 ± 203.2^jk^	133.2 ± 6.7^klmnop^
34	Early Grano	1699.9 ± 178.7^jk^	209.4 ± 11.7^ghij^
35	NHRDF Red-4	1662.2 ± 129.2^jk^	263.3 ± 15.7^fgh^
36	PKV White	1551.9 ± 143.2^jk^	202.4 ± 12.0^hijkl^
37	RO-59	1496.3 ± 27.2^jk^	291.8 ± 10.4^ef^
38	JWO-1	1415.8 ± 123.4^jk^	121.9 ± 8.5^mnopq^
39	Bhima Shubhra	1230.6 ± 13.5^jk^	94.2 ± 6.3^nopq^
40	Talaja Red	1165.1 ± 121.1^k^	292.8 ± 40.5^ef^
41	Bhima Raj	928.3 ± 70.8^k^	83.7 ± 10.9^nopq^
42	Bhima Shweta	892.7 ± 76.7^k^	155.2 ± 13.8^jklmn^
43	GJWO-3	752.7 ± 112.9^k^	112.1 ± 15.2^mnopq^
44	Udaipur Local	732.9 ± 93.4^k^	239.0 ± 23.8^fghi^
45	Agrifound White	490.3 ± 17.0^k^	52.7 ± 3.0^q^

The values are presented as replicated mean ± standard deviation. ^a–q^Means followed by the same letters within a column do not differ significantly.

The K content was recorded to be significantly highest in the yellow-coloured bulb variety from Southern India “Arka Pitamber” (9160.1 ± 96.7 mg/kg of DWB) followed by the Pusa Sona (7933.2 ± 292.8 mg/kg of DWB), GJWO-11 (7875.9 ± 572.8 mg/kg of DWB), and PRO-6 (7690.0 ± 188.2 mg/kg of DWB) varieties. However, minimum K content was assessed in the white-coloured bulb variety “Agrifound White” (490.3 ± 17.0 mg/kg of DWB) followed by Udaipur Local (732.9 ± 93.4 mg/kg of DWB), GJWO-3 (752.7 ± 112.9 mg/kg of DWB), Bhima Shweta (892.7 ± 76.7 mg/kg of DWB), and Bhima Raj (928.3 ± 70.8 mg/kg of DWB).

It was concluded that yellow-coloured bulb varieties (Arka Pitamber and Pusa Sona) exhibited significantly higher K contents than red and white varieties. Twenty-three varieties showed higher potassium contents than the overall mean (4679.3), while 22 exhibited lower values.

Twelve cultivars, including Arka Pitamber, Pusa Sona, GJWO-11, PRO-6, VL Pyaz, Bhima Safed, Pusa Red, Punjab Naroya, Bhima Dark Red, Pusa Riddhi, Kalyanpur Round Red, and Bhima Super, exhibited K content of more than 7000 mg/kg on DWB, whereas nine cultivars, including RO-59, JWO-1, Bhima Shubhra, Talaja Red, Bhima Raj, Bhima Shweta, GJWO-3, Udaipur Local and Agrifound White, had a DWB content of less than 1500 mg/kg in their bulbs when evaluated under the *trans*-gangetic plain zone of New Delhi conditions.

### 3.2. Sodium (Na) content (mg/kg of DWB)

Like K, Na also exhibited broad variation in its concentrations among the tested genotypes (Table 3). The overall average sodium content in onion bulbs was 229.0 mg/kg of DWB, ranging from 52.7 to 458.2. It was determined that there was a more than eightfold difference observed between the highest and lowest Na content in the bulbs of tested cultivars.

In the reverse trend, like K, the Na content was found to be significantly higher in the dark-red-coloured bulb variety “NHRDF-Red L-28” (458.2 ± 61.7 mg/kg of DWB) followed by XP Red (446.8 ± 17.6), RO-252 (421.9 ± 16.9), and Pusa White Flat (395.2 ± 43.2). However, the lowest content was recorded in the white-coloured bulb variety Agrifound White (52.7 ± 3.0 mg/kg of DWB) followed by Akola Safed (68.8 ± 4.15), JRO-11 (70.2 ± 8.7), Pusa Sona (73.8 ± 13.9), and Bhima Raj (83.7 ± 10.9). Among white varieties, the highest Na content was recorded in Pusa White Flat (395.2 ± 43.2), whereas the lowest was found in Agrifound White (52.7 ± 3.0). Twenty-two varieties possessed higher Na than the overall mean (229.0), while 23 recoded values less than this. On the whole, it was further observed that red-coloured varieties elicited higher sodium contents compared to yellow, brown, and white bulb-coloured varieties on a dry weight basis.

Eleven cultivars, including NHRDF-Red L-28, XP Red, RO-252, Pusa White Flat, JNDWO-085, Pusa Red, B-780, VL Pyaz, GJWO-11, Bhima Shakti and Bhima Super, exhibited a DWB Na content of more than 300 mg/kg, whereas ten cultivars including L-819, GJWO-3, Pusa Riddhi, Bhima Shubhra, Hisar-2, Bhima Raj, Pusa Sona, JRO-11, Akola Safed, and Agrifound White elicited a level of less than 120 mg/kg of DWB.

### 3.3. Potassium and sodium (K/Na) ratio

**FIGURE 1:**
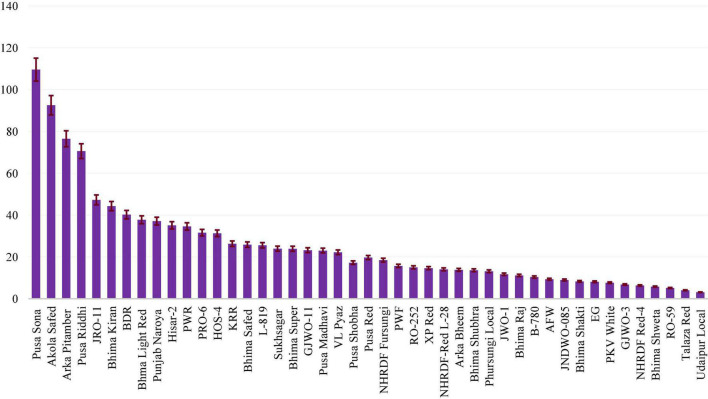
Potassium to sodium ratio in the 45 Indian short day onion cultivars.

The overall average K/Na ratio in onion bulbs was shown to be 25.5, ranging from 3.1 to 109.6. A greater than 35-fold difference was observed between the highest and lowest ratio in the bulbs of tested onion cultivars (Figure 1).

This ratio was significantly higher in the yellow-coloured Northern Indian variety “Pusa Sona” (109.6 ± 17.3) followed by Akola Safed (92.5 ± 7.5), Arka Pitamber (76.5 ± 9.3), Pusa Riddhi (70.6 ± 6.3), and JRO-11 (47.3 ± 6.3). On the other hand, the minimum ratio was estimated in the red-coloured bulb variety from Rajasthan “Udaipur Local” (3.1 ± 0.7) followed by Talaja Red (4.1 ± 0.9), RO-59 (5.1 ± 0.3), Bhima Shweta (5.8 ± 0.9), and NHRDF Red-4 (6.3 ± 0.5). Among the white-coloured bulbs, the maximum ratio was record in Akola Safed (92.6 ± 7.5), while the minimum ratio was reported in GJWO-3 (6.8 ± 1.1). Sixteen varieties showed a higher ratio than the overall mean, while 29 were lower than this value. On the whole, it was further observed that yellow-coloured varieties elicited higher potassium and sodium ratios compared to red, brown, and white bulb-coloured varieties.

Thirteen cultivars, including Pusa Sona, Akola Safed, Arka Pitamber, Pusa Riddhi, JRO-11, Bhima Kiran, Bhima Dark Red, Bhima Light Red, Punjab Naroya, Hisar-2, Pusa White Round, PRO-6 and HOS-4, exhibited a K/Na ratio of more than 30, whereas 11 cultivars, including Agrifound White, JNDWO-085, Bhima Shakti, Early Grano, PKV White, GJWO-3, NHRDF Red-4, Bhima Shweta, RO-59, Talaja Red and Udaipur Local, showed a ratio of less than 10 when evaluated under the *trans*-gangetic plain zone of New Delhi conditions. Varieties (RO-59 and Udaipur Local) selected from Rajasthan, showed a significant yet very low ratio. While two varieties released by IARI, New Delhi *viz*., Pusa White Flat (15.7 ± 2.1) and Pusa White Round (34.6 ± 3.8), showed highly significant differences, almost twofold differences were obtained, these varied depending on the shape of the bulbs. This confirmed that the shape of bulbs is also associated with the K/Na ratio.

### 3.4. Impact of bulb colour and region

**FIGURE 2:**
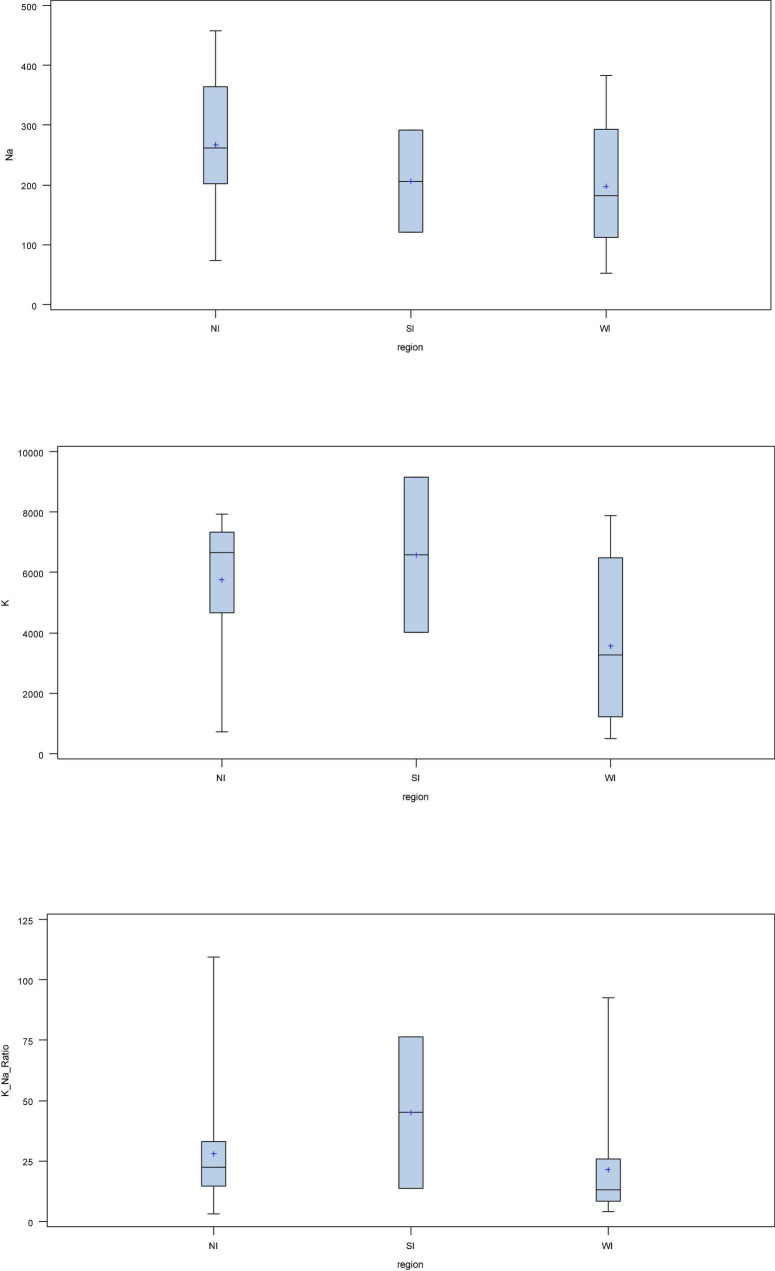
Box plot analysis of K, Na, and K/Na ratio on the basis of region.

**FIGURE 3:**
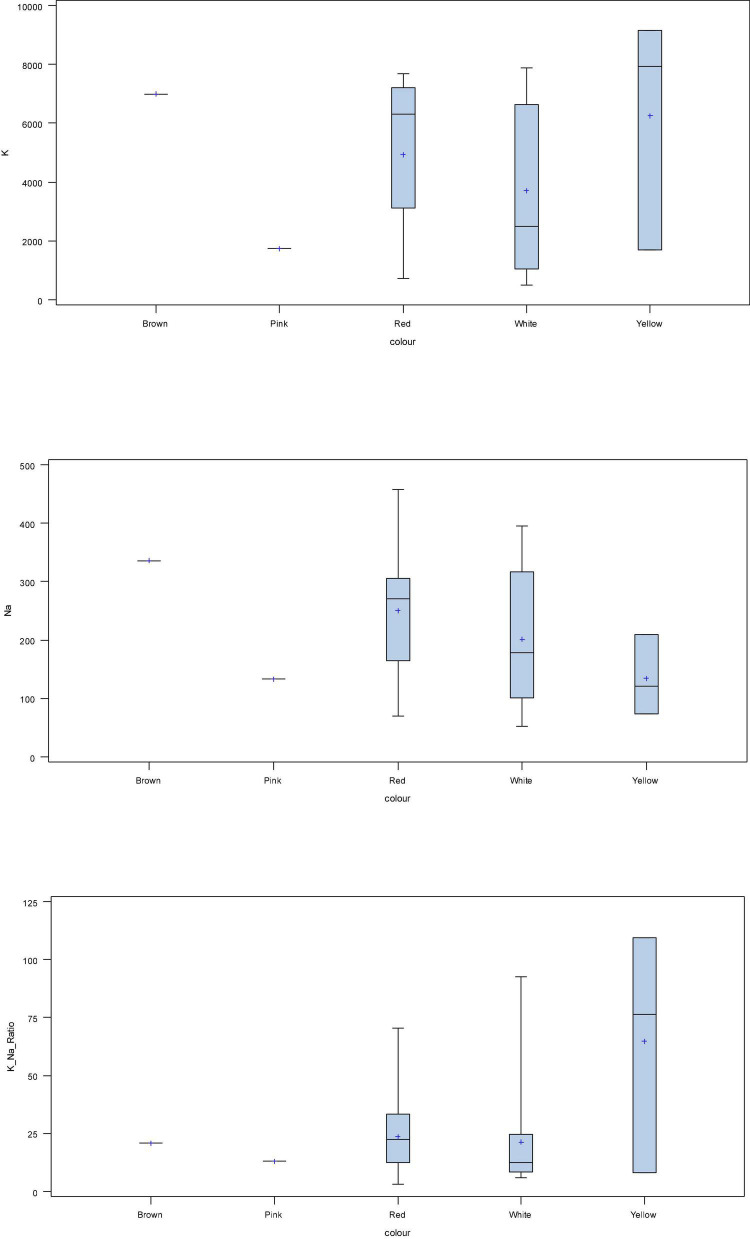
Box plot analysis of K, Na, and K/Na ratio on the basis of bulb colour.

The box plot analysis, based on region, showed that the mean Na and K were highest in the varieties from the North Indian region (NI) followed by South India (SI) and Western India (WI). The highest mean Na/K ratio was observed in onions from SI followed by NI and WI grown onions (Figure 2). In terms of colour, the highest Na was found in brown onions, followed by red-, white-, pink-, and yellow-coloured onions. The highest K was observed in yellow-coloured onions, followed by brown, red, white, and pink onions. The highest mean Na/K ratio was observed in yellow onion, followed by red, brown, pink, and white onion bulbs (Figure 3).

### 3.5. Cluster analysis

**FIGURE 4:**
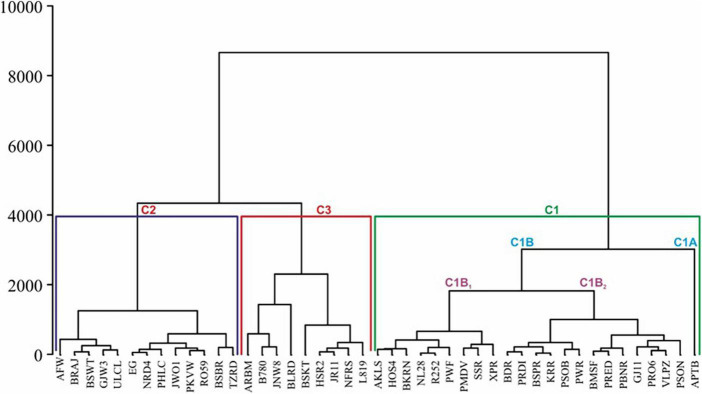
Hierarchical Clustering of 45 genotypes for K, Na, and P/Na ratio based on the distant neighbour and dissimilarities.

The Hierarchical Clustering of all the genotypes was done based on distant neighbours or dissimilarities. The dendrogram is presented in Figure 4. The genotypes grouped in clusters and sub-clusters are presented in Table 4. The dendrogram exhibited three major clusters including 23, 13, and 9 genotypes in cluster C1, C2, and C3, respectively. The cluster I was divided into two groups; group C1A and C1B. The group C1A contained one genotype, i.e., Arka Pitamber which is a yellow-coloured bulb variety. The cluster C1B again subdivided into two categories. The cluster C2 grouped 13 genotypes and divided into two categories included 5 and 8 genotypes. The third cluster C3 contained nine genotypes and mainly consisted of red coloured bulb varieties except JNDWO-085. Except few, most of the northern Indian cultivars grouped into cluster C1.

**TABLE 4:** Cluster analysis of 45 short day Indian onion genotypes for sodium, potassium contents, and potassium/sodium ratio.

Cluster 1 (23) *			Cluster 2 (13)		Cluster 3 (9)	
**1A (1)**	**1B (22)**		**2A (5)**	**2B (8)**	**3A (4)**	**3B (5)**
	**1B_1_ (9)**	**1B_2_ (13)**				
Arka Pitamber	Akola Safed	Bhima Dark Red	AFW	EG	Arka Bheem	Bhima Shakti
	HOS-4	Pusa Riddhi	Bhima Raj	NHRDF Red-4	B-780	Hisar-2
	Bhima Kiran	Bhima Super	Bhima Shweta	Phursungi Local	JNDWO-085	JRO-11
	NHRDF Red L-28	Kalyanpur Red	GJWO-3	JWO-1	BLR	NHRDF Fursungi
	RO-252	Pusa Shobha	Udaipur Local	PKV White		L-819
	Pusa White Flat	Bhima Safed		RO-59		
	Pusa Madhavi	Pusa Red		Bhima Shubhra		
	Sukhsagar	PB Naroya		Talaja Red		
	XP Red	GJWO-11				
		PRO-6				
		VL Pyaz				
		Pusa Sona				
		Pusa White Round				

*Number of genotypes grouped.

## 4. Discussion

Across the globe, people are facing major health issues in the form of metabolic disorders and chronic NCDs. Coronary artery and cerebrovascular (heart stroke) are the most prevalent among cardiovascular diseases (26) and the frequency of deaths due to CVDs increased significantly (27). HTN was found to be the major risk factor (28–30) for CVDS. The intake of higher amounts of sodium and lower amounts of potassium is one of the main reasons for HTN. There is strong evidence that increasing dietary potassium intake reduces both systolic and diastolic blood pressure. Intake of potassium-enriched diets not only reduces blood pressure, but also the risk factors for various CVD, even in elderly and obese subjects with HTN (31–37). A renowned Canadian physician stated that the “*prevalence of arterial hypertension on this continent is in large part due to potash poor diet and an excessive use of salt”* (38). Current diets contain lower potassium (70–80 mmol/day) and higher sodium (150–200 mmol/day), whereas ancestral diets contained much higher potassium (230–300 mmol/day) and negligible (1–10 mmol/day) sodium (39, 40). With the advancements in diet and lifestyle, a major sustainable change was observed after using artificial salt in cooking led to reduction in dietary intake of potassium (31, 41, 42).

In a recent study, Bibbins-Domingo et al. (43) estimated that a reduction in dietary sodium intake of only 1,200 mg per day would reduce the number of stroke cases in the USA from 32,000 to 66,000. Now, it has been scientifically established that a reduction in dietary sodium intake can reduce the risk factors for various CVDs (44). Furthermore, according to the WHO, K is important for blood pressure regulation in hypertensive persons and recommends at least 3510 mg of potassium per day to maintain blood pressure and reduce the risk of CVD. A meta-analysis of 33 randomised controlled trials concluded that potassium supplementation led to a significant reduction in mean systolic and diastolic blood pressure of 3.1 and 2 mmHg, respectively (45).

As an enriched source of dietary K, along with other beneficial bioactive compounds, onion bulbs could be a potential source for hypertensive people to reduce their elevated blood pressure. In low- and middle-income countries, rapid demographic growth and the lower availability of resources have become essential to create cheaper plant-based functional foods that could replace high-cost allopathic medicines, avoid their side effects and ensure nutritional security. Being a versatile crop, onion bulbs are the best option for Indian people since this crop is used in almost all Indian kitchens. Since antiquity, various physicians have prescribed this crop to prevent various ailments and diseases, well documented in the historical literature. India is also a global leader in onion production. Wide genotypic variation in potassium and sodium concentrations and their ratios was recorded in the Indian short day onion bulbs. Our results were supported by the findings of Metrani et al. (17) in the USA. They estimated 12720.7 and 13550.1 mg/kg of DWB K concentrations in red onion long day varieties. However, they found significant differences in the concentration of Na between the two genotypes: 314.1 and 1001.3 mg/kg of DWB. This shows that concentrations of Na and K are highly dependent on the genotype and growing environmental conditions. In onion, growing environmental conditions, agronomic management and genetic makeup of the cultivars determined the variation minerals composition in bulbs.

On the basis of bulb colour, yellow varieties recorded the highest K contents. Varieties like Arka Pitamber and Pusa Sona could be recommended to the Indian population after clinical assessment and bioavailability studies. Twenty-three varieties showed higher potassium contents than the overall mean (4679.3), which clearly showed that the Indian onion population has a higher K content. The major reason of this variability is likely to be due to the genotypic makeup of the cultivar (46). However, this aspect was not even considered for the exploration of a potential source of dietary K. Our results suggest that onion bulbs may offer a sufficient amount of potassium to meet the recommended dietary allowance (RDA) for humans. However, there is no clear-cut RDA for potassium (47).

The human body requires traces of sodium for some metabolic functions, but the consumption of too much sodium results in elevated blood pressure (1), eventually leading to CVD or heart failure. In the current scenario, the identification of plant-based food with low sodium contents is important for decreasing or minimising the risk of CVD and heart attacks. In the present study, an overall average of 229.0 mg/kg of DWB, ranging from 52.7 to 458.2 was observed. Much higher differences were determined between the highest and lowest values. On the contrary, Na content was significantly highest in the dark-red-coloured variety, while K was highest in yellow-coloured onions. Here, our interest is to identify the genotype with low sodium and high potassium contents for further breeding programs. Eleven cultivars exhibited Na content of more than 300 mg/kg of DWB, whereas ten cultivars recorded values of less than 120. Being versatile, with the peculiar flavour of Indian onions, no commercial hybrid is there at national level from the public sector. Cultivators mostly grow open pollinated varieties, so this study might be useful for breeders aiming to develop hybrids with higher contents of potassium using identified genotypes as parents (8).

Various scientific studies have proven that unhealthy diets and HTN play a chief role in the development of various heart diseases. The intake of higher dietary salt and the lower intake of vegetables and fruits are directly associated with a higher risk of CVDs (41, 48) because of elevated blood pressure. The higher intake of potassium and lower intake of sodium is quietly helpful for regulating blood pressure and decreasing the risk of CVDs, especially in hypertensive adults (44, 49). Therefore, the WHO recommended reducing the intake of sodium to less than 2000 mg per day (50) and significantly enhancing the intake of potassium in the diet to a minimum of 3510 mg per day to reduce blood pressure (51). Sodium is usually considered responsible for enhancing blood pressure while potassium antagonistically acts to keep blood pressure within the desired range. Instead of looking at these two elements distinctly, the ratio of the two in the diet has greater significance than the amount of either one alone. Interestingly, the recorded data pertaining to this ratio on the dry weight basis exhibited significant differences and it was further determined that there was a more than 35-fold difference observed between the highest and lowest values.

The K/Na ratio was highest in the yellow-coloured bulb varieties than in red and white onions (Table 5). Sixteen varieties showed higher ratios than the overall mean, while 29 were lower than this. Despite that, the excessive intake of any mineral may prevent other mineral elements from being properly absorbed and utilised in the body. Therefore, the K/Na ratio in onion bulbs is more important to avoid any imbalance. Results of the current research work provide beneficial preliminary information for nutritionists and dieticians involved in developing diet plans and potassium-restricted meals for hypertensive individuals.

**TABLE 5:** Potassium/sodium ratio (K/Na ratio) in short-day Indian OP commercial varieties grown under the *trans*-gangetic plains of India.

S. No	Variety name	K/Na ratio ± SD
1	Pusa Sona	109.6 ± 17.3^a^
2	Akola Safed	92.5 ± 7.5^b^
3	Arka Pitamber	76.5 ± 9.3^c^
4	Pusa Riddhi	70.6 ± 6.3^c^
5	JRO-11	47.3 ± 6.3^d^
6	Bhima Kiran	44.3 ± 3.1^de^
7	Bhima Dark Red	40.2 ± 4.6^def^
8	Bhima Light Red	37.7 ± 4.7^efg^
9	Punjab Naroya	37.1 ± 8.0^efg^
10	Hisar-2	35.1 ± 5.6^fg^
11	Pusa White Round	34.6 ± 3.8^fg^
12	PRO-6	31.6 ± 2.4^gh^
13	HOS-4	31.3 ± 1.4^gh^
14	Kalyanpur Round Red	26.3 ± 4.0^hi^
15	Bhima Safed	25.9 ± 2.9^hi^
16	L-819	25.5 ± 1.4^hi^
17	Sukhsagar	23.9 ± 3.5^hij^
18	Bhima Super	23.9 ± 2.9^hij^
19	GJWO-11	23.2 ± 2.5^ijk^
20	Pusa Madhavi	23.0 ± 2.8^ijk^
21	VL Pyaz	22.2 ± 2.5^ijkl^
22	Pusa Red	19.7 ± 1.1^ijklm^
23	NHRDF Fursungi	18.4 ± 2.0^ijklmn^
24	Pusa Shobha	17.2 ± 1.3^jklmno^
25	Pusa White Flat	15.7 ± 2.1^klmnop^
26	RO-252	15.0 ± 0.9^lmnopq^
27	XP Red	14.6 ± 0.3^lmnopqr^
28	NHRDF-Red L-28	14.1 ± 2.6^mnopqrs^
29	Arka Bheem	13.8 ± 0.6^mnopqrs^
30	Bhima Shubhra	13.6 ± 0.9^mnopq^
31	Phursungi Local	13.1 ± 2.1^mnopqrst^
32	JWO-1	11.7 ± 1.8^mnopqrstu^
33	Bhima Raj	11.2 ± 0.9^nopqrstuv^
34	B-780	10.4 ± 1.0^nopqrstuv^
35	Agrifound White	9.3 ± 0.7^opqrstuv^
36	JNDWO-085	9.0 ± 0.4^pqrstuv^
37	Bhima Shakti	8.3 ± 0.6^pqrstuv^
38	Early Grano	8.1 ± 0.5^pqrstuv^
39	PKV White	7.7 ± 0.5^pqrstuv^
40	GJWO-3	6.8 ± 1.1^qrstuv^
41	NHRDF Red-4	6.3 ± 0.5^rstuv^
42	Bhima Shweta	5.8 ± 0.9^stuv^
43	RO-59	5.1 ± 0.3^tuv^
44	Talaja Red	4.1 ± 0.9^uv^
45	Udaipur Local	3.1 ± 0.7^v^

The values are presented as replicated mean ± standard deviation. ^a–v^Means followed by the same letters within a column do not differ significantly.

One of the possible contraindications to onion consumption is the FODMAPs- content. FODMAPs are a category of carbs and fibres that many people cannot tolerate. They may cause unpleasant digestive symptoms, such as bloating, gas, cramping, and diarrhoea. Individuals suffering from irritable bowel syndrome are often intolerant to FODMAPs and may need to avoid onions. (52).

## Conclusion

From the findings of the current study, it could be determined that the Indian onion may be considered a potential source of potassium, as well as having low sodium contents. Yellow bulb onions recorded higher potassium levels than red- and white-coloured onions, which may be useful for hypertensive individuals to prevent various CVDs. Further, it should be explored using comprehensive investigations as a potential source of dietary potassium. That information could be used to develop diet plans and further breeding programs to develop specific cultivars for populations with improved potency. Although the comprehensive clinical and physiological implications remain to be established, our findings and information generated on Indian onion further cater to emphasise the need for future studies focused on the development of functional foods as a public health approach. Still, imperative questions with respect to bioavailability and physiological and molecular pathways still need much more attention from plant physiologists. Additionally, molecular level studies for the identification of genotypes with significantly higher potassium-to-sodium ratios will be beneficial for breeders and geneticists aiming to develop new climate smart resilient cultivars with desired quality and nutrition factors. Ultimately, all such novel approaches may help to alleviate HTN effects in the global population, which is an alarming worldwide challenge faced by public health and plant scientists. In the 21st century, dietary interventions to reduce the occurrence of HTN should have immense potential to considerably decrease CVD morbidity and mortality. Furthermore, well-designed clinical trials are required to test the probable effects of various Indian varieties with high potassium-to-sodium ratios on blood pressures and various cardiovascular and cerebrovascular events.

## Data availability statement

The original contributions presented in this study are included in the article/supplementary material, further inquiries can be directed to the corresponding authors.

## Author contributions

HS and AnK: conceptualization, formal analysis, and methodology. HS: investigation and writing – original draft. HS, AnK, and AmK: software. AnK: supervision. HS: writing – original draft. AG, AmK, AnK, HS, and ML: interpretation of data. AnK, ML, and AG: writing – review and editing. All authors read and approved the final manuscript.
