# Corrigendum: Genotypic variation in Na, K and their ratio in 45 commercial cultivars of Indian tropical onion: A pressing need to reduce hypertension among the population

**DOI:** 10.3389/fnut.2023.1182998

**Published:** 2023-03-23

**Authors:** Hira Singh, Mauro Lombardo, Abhishek Goyal, Amrender Kumar, Anil Khar

**Affiliations:** ^1^Department of Vegetable Science, Punjab Agricultural University, Ludhiana, India; ^2^Division of Vegetable Science, ICAR- Indian Agricultural Research Institute, New Delhi, India; ^3^Department of Human Sciences and Promotion of the Quality of Life, San Raffaele Open University, Rome, Italy; ^4^Department of Cardiology, Dayanand Medical College and Hospital, Ludhiana, India; ^5^Agricultural Knowledge Management Unit, ICAR-Indian Agricultural Research Institute, New Delhi, India

**Keywords:** *Allium cepa* L., cardiovascular diseases, hypertension, onion bulbs, potassium, potassium/sodium ratio, sodium

In the published article, there was an error regarding the affiliations for author Hira Singh. As well as having affiliation 1, they should also have Division of Vegetable Science, ICAR-Indian Agricultural Research Institute, New Delhi, India.

An omission to the funding section of the original article was made in error. The following funding statement has been added:

Authors are thankful to the ICAR-Indian Agricultural Research Institute, New Delhi for providing financial support and conduct of the research program of the PhD student, HS. The research work was partially funded by the NAHEP-CAAST programme of Indian Council of Agricultural Research.

The authors apologize for these errors and state that this does not change the scientific conclusions of the article in any way. The original article has been updated.

